# Recently published *Streptomyces* genome sequences

**DOI:** 10.1111/1751-7915.12143

**Published:** 2014-08-07

**Authors:** James Harrison, David J Studholme

**Affiliations:** Biosciences, College of Life and Environmental Sciences, University of ExeterExeter, UK

## Introduction

Many readers of this journal will need no introduction to the bacterial genus *Streptomyces*, which includes several hundred species, many of which produce biotechnologically useful secondary metabolites. The last 2 years have seen numerous publications describing *Streptomyces* genome sequences (Table 1), mostly as short genome announcements restricted to just 500 words and therefore allowing little description and analysis. Our aim in this current manuscript is to survey these recent publications and to dig a little deeper where appropriate. The genus *Streptomyces* is now one of the most highly sequenced, with 19 finished genomic sequences (Table 2) and a further 125 draft assemblies available in the GenBank database as of 3rd of May 2014; by the time this is published, no doubt there will be more. The reasons given for sequencing this latest crop of *Streptomyces* include production of industrially important enzymes, degradation of lignin, antibiotic production, rapid growth and halo-tolerance and an endophytic lifestyle (Table 1).

**Table 1:** Recent genome publications (2013 and 2014) for *Streptomyces* species

Species and strain	Motivation for sequencing
*Streptomyces albulus* CCRC 11814 (Dodd *et al*., 2013)	Produces ε-poly-l-lysine antibiotic.
*Streptomyces albus* J1074 (Zaburannyi *et al*., 2014)	Widely used host for heterologous expression of bioactive natural products. Small genome.
*Streptomyces albulus* PD-1 (Xu *et al*., 2014)	Produces ε-poly-l-lysine and poly-L-diaminopropionic acid antibiotics.
*Streptomyces bottropensis* ATCC 25435 (Zhang *et al*., 2013)	Produces bottromycin antibiotics.
*Streptomyces collinus* Tu 365 (Rückert *et al*., 2013)	Producer the elfamycin-family antibiotic kirromycin.
*Streptomyces exfoliatus* DSMZ 41693 (Martínez *et al*., 2014)	Degrades poly(3-hydroxyalkanoate).
*Streptomyces fulvissimus* DSM 40593 (Myronovskyi *et al*., 2013)	Produces the ionophore antibiotic valinomycin.
*Streptomyces gancidicus* BKS 13–15 (Kumar *et al*., 2013)	Not known.
*Streptomyces mobaraensis* DSM 40847 (Yang *et al*., 2013)	Industrial producer of transglutaminase.
*Streptomyces niveus* NCIMB 11891 (Flinspach *et al*., 2014)	Produces novobiocin, an aminocoumarin antibiotics.
*Streptomyces rapamycinicus* NRRL 5491 (Baranasic *et al*., 2013)	Produces the immunosuppressant drug rapamycin.
*Streptomyces rimosus* ATCC 10970 (Pethick *et al*., 2013)	Oxytetracycline
*Streptomyces roseochromogenes* subsp. *oscitans* DS 12.976 (Rückert *et al*., 2014)	Produces clorobiocin, an aminocoumarin antibiotic.
*Streptomyces* species Mg1 (Hoefler *et al*., 2013)	Causes lysis and degradation of *Bacillus subtilis* cells and colonies. Sequenced using the PacBio platform.
*Streptomyces* species PRh5 (Yang *et al*., 2014)	An endophyte isolated from wild rice root.
*Streptomyces violaceusniger* SPC6 (Chen *et al*., 2013)	Tolerant to multiple stresses. Small genome.
*Streptomyces viridochromogenes* Tu57 (Grüning *et al*., 2013)	Produces the oligosaccharide antibiotic avilamycin.
*Streptomyces viridosporus* T7A (Davis *et al*., 2013)	Degrades lignin.

**Table 2:** Completely sequenced *Streptomyces* species genome sequences available in GenBank as of 29 April 2014

Species and strain	GenBank accession numbers
*Streptomyces albus* J1074 (Zaburannyi *et al*., 2014)	CP004370
*Streptomyces avermitilis* (Omura *et al*., 2001; Ikeda *et al*., 2003)	AP005645, BA000030
*Streptomyces bingchenggensis* BCW 1 (Wang *et al*., 2010)	CP002047
*Streptomyces cattleya* NRRL 8057 (no publication)	CP003219, CP003229
*Streptomyces cattleya* NRRL 8057 (Barbe *et al*., 2011)	FQ859184, FQ859185
*Streptomyces coelicolor* A3(2) (Bentley *et al*., 2002)	AL589148, AL645771, AL645882
*Streptomyces collinus* Tu 365 (Rückert *et al*., 2013)	CP006259, CP006260, CP006261
*Streptomyces davawensis* JCM 4913 (Jankowitsch *et al*., 2012)	HE971709, HE971710
*Streptomyces flavogriseus* ATCC 33331 (no publication)	CP002475, CP002476, CP002477
*Streptomyces fulvissimus* DSM 40593 (Myronovskyi *et al*., 2013)	CP005080
*Streptomyces griseus* NBRC 13350 (Ohnishi *et al*., 2008)	AP009493
*Streptomyces hygroscopicus jinggangensis* 5008 (Wu *et al*., 2012)	CP003275, CP003276, CP003277
*Streptomyces hygroscopicus jinggangensis* TL01 (no publication)	CP003720, CP003721, CP003722
*Streptomyces* sp. PAMC26508 (no publication)	CP003990, CP003991
*Streptomyces rapamycinicus* NRRL 5491 (Baranasic *et al*., 2013)	CP006567
*Streptomyces scabiei* 87 22 (Bignell *et al*., 2010)	FN554889
*Streptomyces* sp. SirexAA E (no publication)	CP002993
*Streptomyces venezuelae* ATCC 10712 (Pullan *et al*., 2011)	FR845719
*Streptomyces violaceusniger* Tu 4113 (no publication)	CP002994, CP002995, CP002996

## Mining genomes for secondary metabolism gene clusters

Given the strong emphasis on secondary metabolism in *Streptomyces* genomics research, it is timely that version 2.0 of antiSMASH has been released and published (Blin *et al*., 2013). This computational tool has become a *de facto* standard for mining secondary metabolism gene clusters in genome sequences. Version 2.0 is completely revamped and, significantly, can now be used with highly fragmented draft-quality genome sequences whereas the previous version only worked well with finished genomes. Clearly, this is of immense importance to the discovery of novel metabolites in the ever-expanding database of streptomycete draft-quality genome sequences. For example, antiSMASH 2.0 analysis of the *Streptomyces roseochromogenes* subsp. *oscitans* DS 12.976 genome sequence revealed 43 new gene clusters in addition to recovering the already known clorobiocin gene cluster (Rückert *et al*., 2014).

The genome sequence of *Streptomyces gancidicus* strain BKS 13–15 was published before antiSMASH 2.0 became available. The authors state that seven genes mapped on to the streptomycin biosynthesis pathway based on gene-by-gene sequence similarities (Kumar *et al*., 2013) against homologues of genes in KEGG pathways (Kanehisa *et al*., 2012). However, we found no bioinformatic evidence for a streptomycin biosynthesis pathway encoded in this genome, although our antiSMASH 2.0 search did find 38 putative gene clusters. In common with many other pathways for secondary metabolism, genes for production of the aminoglycoside streptomycin are organized into a cluster of contiguous genes. The nucleotide sequences of at least two such clusters are available (GenBank accessions GU384160 and AJ862840 from *Streptomyces platensis* and *Streptomyces griseus* respectively). Our blastn searches (using these two cluster sequences as queries) failed to detect a complete streptomycin gene cluster in the *S. gancidicus* genome, but there were some regions of sequence similarity on a 111 kb contig (GenBank: AOHP01000057). An antiSMASH 2.0 search failed to find any aminoglycoside biosynthetic cluster in this genome. We are not aware of any experimental evidence that this strain produces the aminoglycoside streptomycin and conclude that these seven genes highlighted by the authors (Kumar *et al*., 2013) most probably encode components of another, perhaps novel, pathway. This illustrates the value of the antiSMASH 2.0 tool, which has the potential to discover new pathways, rather than relying on similarity to the pathways already represented in the KEGG database (and therefore, by definition, not novel).

**Figure 1:**
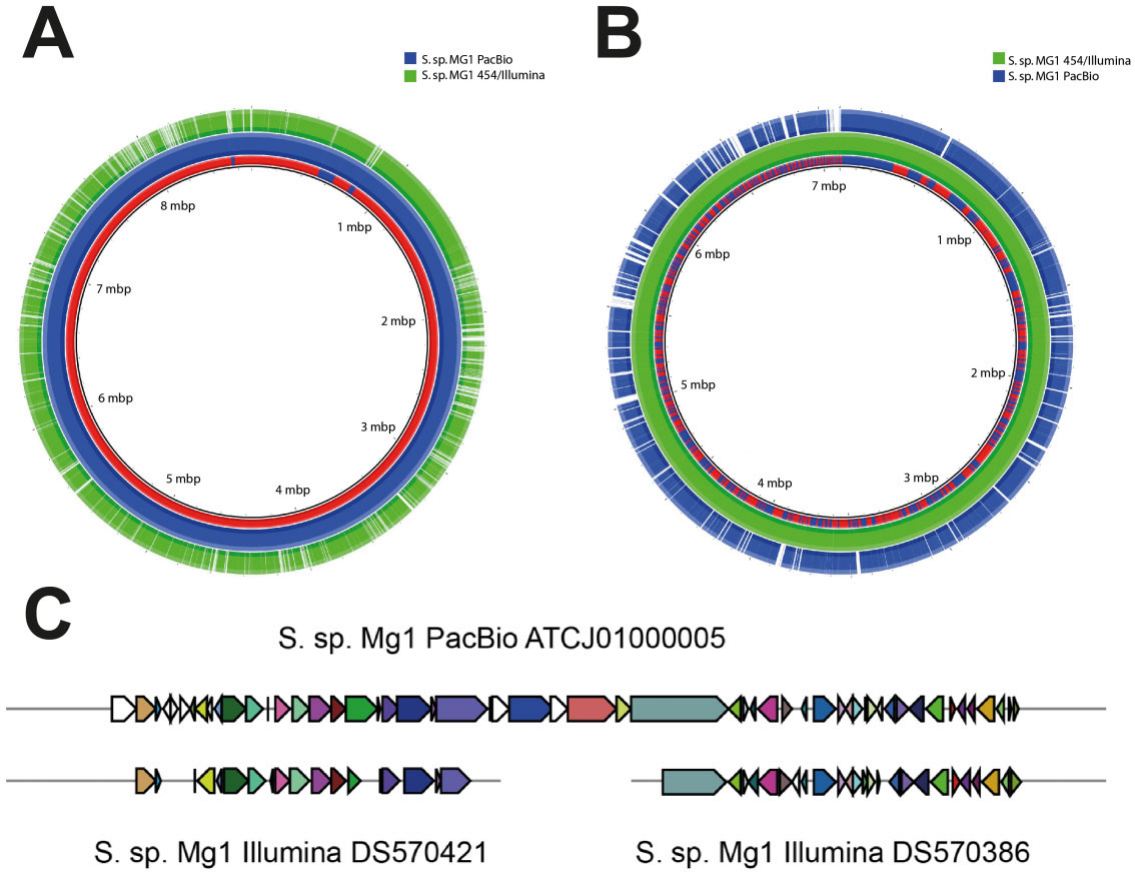
Comparison of two different genome assemblies for *Streptomyces* strain Mg1, one based on PacBio sequence data and the other based on 454 and Illumina sequence data. A illustrates alignment of both the assemblies against the PacBio-based assembly. B illustrates both the assemblies aligned against the 454/Illumina-based assembly. C illustrates a novel secondary-metabolism gene cluster identified by antiSMASH 2.0 (Blin *et al*., 2013) in both assemblies. The entire cluster is recovered intact in the PacBio-based assembly but it is split across two different contigs in the 454/Illumina-based assembly and part of the middle of the cluster is missing. Alignments in A and B were generated using Basic Local Alignment Search Tool Nucleotide tool blastn (Altschul *et al*., 1990) and visualized using the blast Ring Image Generator (BRIG) (Alikhan *et al*., 2011). The innermost ring indicates the genomic position. The next ring is a plot of G + C content. The remaining five concentric rings indicate the presence or absence of blastn hits at that position, with one ring corresponding to each of the five indicated genome assemblies. To aid clarity, each ring is represented in a different colour. Positions covered by blastn alignments are indicated with a solid colour; whitespace gaps represent genomic regions not covered by the blastn alignments. The graphics in C were cut and pasted directly from the antiSMASH output.

The case of *Streptomyces* species strain Mg1 (Hoefler *et al*., 2013) illustrates another consideration when mining bacterial genome sequences for secondary metabolism gene clusters. Many of the recently published *Streptomyces* genome sequences are assembled from massively parallel sequencing platforms such as 454 GS-FLX and Illumina HiSeq. The short sequence reads (typically less than 450 bp) and relatively high error rates associated with these platforms can lead to rather fragmented and/or incomplete genome assemblies. The situation is not helped by the biased sequence composition (approximately 70% G + C) of *Streptomyces* DNA. Furthermore, non-ribosomal peptide synthases (NRPS) and polyketide synthetases (PK) are long, modular proteins made up of many repeated domain units. This means that the genes encoding these key enzymes can be particularly difficult to assemble accurately from short sequence reads. To overcome this issue, the authors of the Mg1 genome project (Hoefler *et al*., 2013) exploited the PacBio SMRT sequencing technology, which provides sequences reads of several Kb in length, meaning that an entire PK or NRPS gene could be represented on a single sequence read, thus avoiding the difficulties of assembling repetitive sequence from short fragments. They also generated an assembly of the same genome based on 454 GS-FLX and Illumina HiSeq. The results were striking: more than 90% of the genome was represented in a single contig of 7.8 Mb in the PacBio-based assembly and the PacBio-based assembly was 19.9% longer than the 454/Illumina-based one (8 705 754 versus 7 260 368 bp). As the authors point out, this implies that more than 1 Mb of sequence in the PacBio-based assembly is missing from the 454/Illumina-based one, as can be seen in Fig. 1A. However, the 454/Illumina-based assembly is not simply a subset of the PacBio-based one; as illustrated in Fig. 1B, a substantial portion of the 454/Illumina-based assembly is missing from the PacBio assembly. Although it is by no means certain which assembly is more ‘correct’, it might be possible to generate a more complete genome assembly by reconciling the two different assemblies.

Fragmentation and incompleteness of a genome assembly has implications for discovery of secondary metabolism gene clusters. In Fig. 1C, we show a putative NRPS gene cluster detected apparently intact in a single contig of the PacBio-based sequence assembly identified by antiSMASH 2.0. Searching the 454/Illumina-based assembly reveals two incomplete fragments of the gene cluster, lying on two different contigs, and with part of the cluster apparently absent. Although we should be cautious about extrapolating too much from this single anecdotal example, the evidence suggests that longer read lengths can be very valuable in genome mining for secondary metabolism clusters.

## Digesting wood: *Streptomyces viridosporus* T7A

Streptomycetes may have important applications other than production of secondary metabolites, for example lignin degradation (Thomas and Crawford, 1998; Bugg *et al*., 2011; Brown and Chang, 2014). The aromatic polymer lignin is a major component of plant material and there is significant interest in organisms that can break down lignocellulose waste materials to generate useful products such as bioethanol (Bugg *et al*., 2011). Digestion of lignin is important not only because it can comprise up to 30% of plant biomass but also because its removal is necessary to facilitate degradation of hemicellulose and cellulose. The enzymology of lignin degradation is best understood in fungi, but it has become apparent that a number of bacterial species also have this capability (Brown and Chang, 2014). For example, *S. viridosporus* T7A is able to solubilize lignin, probably via the action of at least one extracellular peroxidase (Thomas and Crawford, 1998). A complete genome sequence is now available for this strain (Davis *et al*., 2013), revealing a number of genes encoding candidate lignin-degrading enzymes (see Table 3). This species is closely related to *Streptomyces ghanaensis* for which a genome sequence is also available (GenBank: ABYA00000000) and which is notable for its production of the antibiotic moenomycin A (Subramaniam-Niehaus *et al*., 1997; Ostash *et al*., 2007; 2009). Most of the candidate lignin metabolism genes in Table 3 are also conserved in *S. ghanaensis*. We are not aware of any published reports of *S. ghanaensis* being able to degrade lignin, but it would be interesting to experimentally test whether it has this capability; if it does not, then comparative genomics between these closely related strains might reveal novel genetic determinants of lignin degradation.

**Table 3:** Candidate genes for involvement in lignin degradation in *Streptomyces viridosporus* T7A

Genomic location (GenBank accession and start–end positions)	Predicted function
JH993790.1: 2305800-2307098	Dyp-type peroxidase family protein
JH993790.1: 622584-623462	Catechol 12C2-dioxygenase 1 (EC: 1.13.11.1)
JH993790.1: 6059025-6059630	3-oxoadipate enol-lactone hydrolase/4-carboxymuconolactone decarboxylase
JH993790.1: 6059861-6061066	Acetyl-CoA acetyltransferase (EC: 2.3.1.9)
JH993790.1: 6061063-6061707	Succinyl-CoA:3-ketoacid-coenzyme A transferase subunit B (EC: 2.8.3.5)
JH993790.1: 6062462-6062758	Muconolactone isomerase (EC: 5.3.3.4)
JH993790.1: 6062765-6063619	Catechol 12C2-dioxygenase (EC: 1.13.11.1)
JH993790.1: 6063652-6064740	Muconate cycloisomerase (EC: 5.5.1.1)
JH993790.1: 6064752-6065651	Aromatic hydrocarbon utilization transcriptional regulator CatR (LysR family)
JH993790.1: 6065818-6067236	Benzoate 12C2-dioxygenase alpha subunit (EC: 1.14.12.10)
JH993790.1: 6067233-6067739	Benzoate 12C2-dioxygenase beta subunit (EC: 1.14.12.10)
JH993790.1: 6067770-6068810	Benzoate dioxygenase2C ferredoxin reductase component
JH993790.1: 6068807-6069583	benzoate dioxygenase2C ferredoxin reductase component / 12C2-dihydroxycyclohexa-32C5-diene-1-carboxylate dehydrogenase (EC: 1.3.1.25)
JH993790.1: 6069731-6071113	Benzoate MFS transporter BenK
JH993790.1: 6073974-6075281	Benzoate transport protein
JH993789.1: 1053498-1054280	Succinyl-CoA:3-ketoacid-coenzyme A transferase subunit A (EC: I6683)
JH993789.1: 1054280-1054924	Succinyl-CoA:3-ketoacid-coenzyme A transferase subunit B (EC: 2.8.3.5)
JH993789.1: 1054921-1055700	Protocatechuate 32C4-dioxygenase beta chain (EC: 1.13.11.3)
JH993789.1: 1055707-1056312	Protocatechuate 32C4-dioxygenase alpha chain (EC: 1.13.11.3)
JH993789.1: 1056309-1057640	3-carboxy-cis2Ccis-muconate cycloisomerase (EC: 5.5.1.2)
JH993789.1: 1057637-1058767	4-carboxymuconolactone decarboxylase (EC: 4.1.1.44)
JH993789.1: 337401-338228	Non-heme chloroperoxidase (EC: 1.11.1.10)

## Genome size: *Streptomyces violaceusniger*

**Figure 2:**
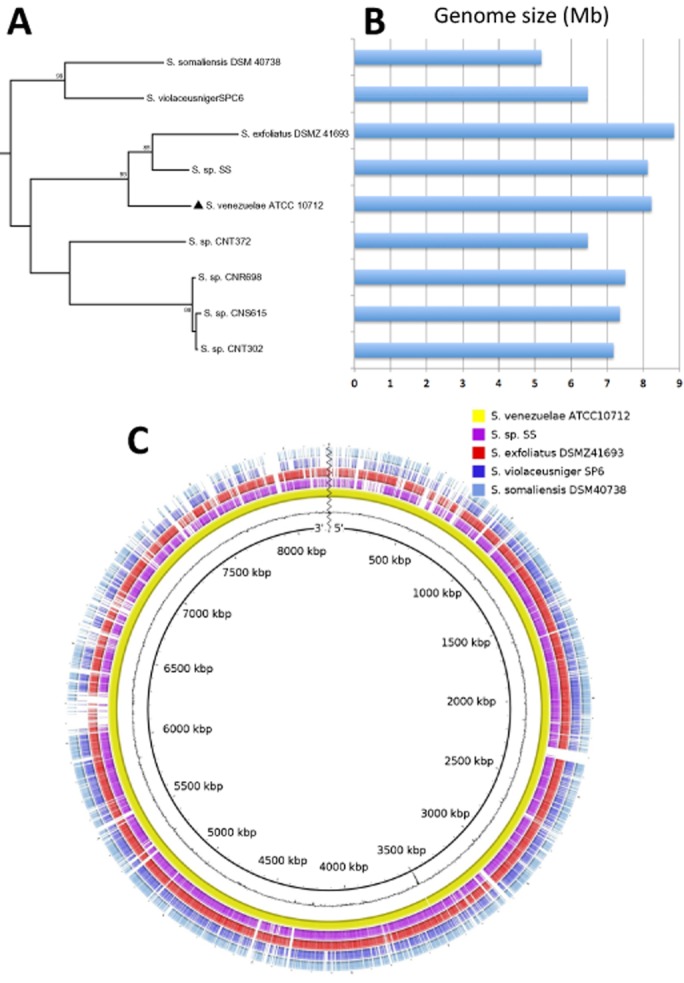
Variation in genome size among *Streptomyces somaliensis* and its close relatives. A shows a section of a maximum-likelihood phylogenetic tree based on aligned sequences of five housekeeping genes (*atpD*, *gyrB*, *recA*, *rpoB*, *trpB*) extracted from draft genome sequence assemblies or, in the case of *S. venezuelae*, finished genome sequence, which is indicated by the black triangle. The tree was generated using MEGA6 (Tamura *et al*., 2013). B indicates the length of each genome assembly. C illustrates alignments of each genome assembly against the *S. venezuelae* reference genome, which consists of a single linear chromosome. Alignments were generated using Basic Local Alignment Search Tool Nucleotide tool blastn (Altschul *et al*., 1990) and visualized using the blast Ring Image Generator (BRIG) (Alikhan *et al*., 2011). The innermost ring indicates the genomic position. The next ring is a plot of G + C content. The remaining five concentric rings indicate the presence or absence of blastn hits at that position, with one ring corresponding to each of the five indicated genome assemblies. To aid clarity, each ring is represented in a different colour. Positions covered by blastn alignments are indicated with a solid colour; whitespace gaps represent genomic regions not covered by the blastn alignments.

Among bacteria, streptomycetes have some of the largest genomes, typically within the range of 8.7 Mbp to 11.9 Mbp (Zhou *et al*., 2012). However, the recently reported genome sequence of *S. violaceusniger* strain SP6 weighs in at just 6.4 Mb (Chen *et al*., 2013) and that of *Streptomyces albus* J1074 6.8 Mb (Zaburannyi *et al*., 2014). Although both sets of authors (Chen *et al*., 2013; Zaburannyi *et al*., 2014) claim theirs as the smallest reported genome of any streptomycete, in fact that record is held by the previously sequenced *Streptomyces somaliensis* strain DSM 40738, a pathogenic strain isolated from a human infection (Kirby *et al*., 2012). The assembly of this genome was just 5.18 Mbp in length; the authors of that study claim that this is consistent with results from pulsed-field gel electrophoresis. Our multilocus sequence analysis (data not shown) reveals that strain SPC6, also known as *Streptomyces thermolilacinus* SPC6, is not closely related to *S. violaceusniger* strain TU 4113 (GenBank: CP002994), which has an 11.14 Mbp genome. Rather, strains SPC6 and DSM 40736 are closely related and fall within a clade with several other strains for which draft genomes are available and with *Streptomyces venezuelae* for which a complete finished genome sequence is available (Pullan *et al*., 2011). Figure 2 shows the sizes of these genomes. It appears that genome reduction may have occurred at least twice in this clade: once in a common ancestor of SPC6 and DSM 40738, and also independently in an ancestor of strain CNT372 (GenBank: ARHT00000000). It is even possible that genome reduction has occurred independently in SPC6 and DSM 40738 as Fig. 2C reveals differences as well as similarities in gene conservation with respect the *S. venezuelae* reference sequence. Evidently, genome reduction has also occurred in *S. albus* strain J1074 (Zaburannyi *et al*., 2014), which is not closely related to this clade. In this strain, the reduction seems to have been achieved by deletion of duplicated genes. The evolutionary driver for genome reduction in streptomycetes is unclear, although it might not be mere coincidence that the smallest genome reported so far is from a pathogen, namely *S. somaliensis* (Kirby *et al*., 2012), and evolution of pathogenesis is often associated with genome reduction (Toft and Andersson, 2010).

## The future of *Streptomyces* genomics

The availability of cheap sequencing has led to the generation of numerous genome sequences for *Streptomyces* and related species [e.g. (Liu *et al*., 2013)] with the objective of discovering novel metabolic products. However, sequencing the genome and discovering novel gene clusters is just the beginning; many of the metabolic products of these gene clusters are ‘cryptic’, not being expressed under normal laboratory conditions. Productive ‘genome mining’ requires either genetic modification of the cluster to force expression or cloning and expression of the cluster in a heterologous host (Gomez-Escribano and Bibb, 2014). The value of this approach, even starting from rather poor-quality draft genome sequences, has been demonstrated by the discovery of the gene cluster encoding cypemycin in *Streptomyces* sp. strain OH-4156, revealing an unusual class of post-translationally modified ribosomally synthesized peptides (Claesen and Bibb, 2010). There will inevitably be a lag between the initial frenzy of genome sequencing and the characterization of novel useful products as the biochemical investigations are more laborious than the sequencing. Another interesting emerging theme is the role of endophytic streptomycetes and the emerging picture that secondary metabolites contribute to the medicinal properties of their host plants [e.g. (Akshatha *et al*., 2014)]. The most recently published *Streptomyces* genome comes from strain PRh5, an endophyte of wild rice that produces nigericin, an antibiotic effective against mycobacteria (Yang *et al*., 2014).

## References

[b1] Akshatha VJ, Nalini MS, D’Souza C, Prakash HS (2014). Streptomycete endophytes from anti-diabetic medicinal plants of the Western Ghats inhibit alpha-amylase and promote glucose uptake. Lett Appl Microbiol.

[b2] Alikhan N-F, Petty NK, Ben Zakour NL, Beatson SA (2011). BLAST Ring Image Generator (BRIG): simple prokaryote genome comparisons. BMC Genomics.

[b3] Altschul SF, Gish W, Miller W, Myers EW, Lipman DJ (1990). Basic local alignment search tool. J Mol Biol.

[b4] Baranasic D, Gacesa R, Starcevic A, Zucko J, Blazic M, Horvat M (2013). Draft genome sequence of *Streptomyces rapamycinicus* strain NRRL 5491, the producer of the immunosuppressant rapamycin. Genome Announc.

[b5] Barbe V, Bouzon M, Mangenot S, Badet B, Poulain J, Segurens B (2011). Complete genome sequence of *Streptomyces cattleya* NRRL 8057, a producer of antibiotics and fluorometabolites. J Bacteriol.

[b6] Bentley SD, Chater KF, Cerdeño-Tárraga A-M, Challis GL, Thomson NR, James KD (2002). Complete genome sequence of the model actinomycete *Streptomyces coelicolor* A3(2). Nature.

[b7] Bignell DRD, Seipke RF, Huguet-Tapia JC, Chambers AH, Parry RJ, Loria R (2010). *Streptomyces scabies* 87–22 contains a coronafacic acid-like biosynthetic cluster that contributes to plant-microbe interactions. Mol Plant Microbe Interact.

[b8] Blin K, Medema MH, Kazempour D, Fischbach MA, Breitling R, Takano E, Weber T (2013). antiSMASH 2.0–a versatile platform for genome mining of secondary metabolite producers. Nucleic Acids Res.

[b9] Brown ME, Chang MC (2014). Exploring bacterial lignin degradation. Curr Opin Chem Biol.

[b10] Bugg TDH, Ahmad M, Hardiman EM, Singh R (2011). The emerging role for bacteria in lignin degradation and bio-product formation. Curr Opin Biotechnol.

[b11] Chen X, Zhang B, Zhang W, Wu X, Zhang M, Chen T (2013). Genome sequence of *Streptomyces violaceusniger* strain SPC6, a halotolerant streptomycete that exhibits rapid growth and development. Genome Announc.

[b12] Claesen J, Bibb M (2010). Genome mining and genetic analysis of cypemycin biosynthesis reveal an unusual class of posttranslationally modified peptides. Proc Natl Acad Sci USA.

[b13] Davis JR, Goodwin L, Teshima H, Detter C, Tapia R, Han C (2013). Genome sequence of *Streptomyces viridosporus* strain T7A ATCC 39115, a lignin-degrading actinomycete. Genome Announc.

[b14] Dodd A, Swanevelder D, Featherston J, Rumbold K (2013). Draft Genome sequence of *Streptomyces albulus* strain CCRC 11814, an ε-poly-L-lysine-producing actinomycete. Genome Announc.

[b15] Flinspach K, Rückert C, Kalinowski J, Heide L, Apel AK (2014). Draft genome sequence of *Streptomyces niveus* NCIMB 11891, producer of the aminocoumarin antibiotic novobiocin. Genome Announc.

[b16] Gomez-Escribano JP, Bibb MJ (2014). Heterologous expression of natural product biosynthetic gene clusters in *Streptomyces coelicolor*: from genome mining to manipulation of biosynthetic pathways. J Ind Microbiol Biotechnol.

[b17] Grüning BA, Erxleben A, Hähnlein A, Günther S (2013). Draft genome sequence of *Streptomyces viridochromogenes* strain Tu57, producer of avilamycin. Genome Announc.

[b18] Hoefler BC, Konganti K, Straight PDP (2013). De novo assembly of the *Streptomyces* sp. strain Mg1 genome using PacBio single-molecule sequencing. Genome Announc.

[b19] Ikeda H, Ishikawa J, Hanamoto A, Shinose M, Kikuchi H, Shiba T (2003). Complete genome sequence and comparative analysis of the industrial microorganism *Streptomyces avermitilis*. Nat Biotechnol.

[b20] Jankowitsch F, Schwarz J, Rückert C, Gust B, Szczepanowski R, Blom J (2012). Genome sequence of the bacterium *Streptomyces davawensis* JCM 4913 and heterologous production of the unique antibiotic roseoflavin. J Bacteriol.

[b21] Kanehisa M, Goto S, Sato Y, Furumichi M, Tanabe M (2012). KEGG for integration and interpretation of large-scale molecular data sets. Nucleic Acids Res.

[b22] Kirby R, Sangal V, Tucker NP, Zakrzewska-Czerwinska J, Wierzbicka K, Herron PR (2012). Draft genome sequence of the human pathogen *Streptomyces somaliensis*, a significant cause of actinomycetoma. J Bacteriol.

[b23] Kumar S, Kaur N, Singh NNK, Raghava GPS, Mayilraj S (2013). Draft genome sequence of *Streptomyces gancidicus* strain BKS 13–15. Genome Announc.

[b24] Liu W-B, Yu W-B, Gao S-H, Ye B-C (2013). Genome sequence of saccharopolyspora erythraea D, a hyperproducer of erythromycin. Genome Announc.

[b25] Martínez V, Hormigo D, del Cerro C, Gómez de Santos P, García-Hidalgo J, Arroyo M (2014). Genome sequence of *Streptomyces exfoliatus* DSMZ 41693, a source of Poly(3-Hydroxyalkanoate)-Degrading enzymes. Genome Announc.

[b26] Myronovskyi M, Tokovenko B, Manderscheid N, Petzke L, Luzhetskyy A (2013). Complete genome sequence of *Streptomyces fulvissimus*. J Biotechnol.

[b27] Ohnishi Y, Ishikawa J, Hara H, Suzuki H, Ikenoya M, Ikeda H (2008). Genome sequence of the streptomycin-producing microorganism *Streptomyces griseus* IFO 13350. J Bacteriol.

[b28] Omura S, Ikeda H, Ishikawa J, Hanamoto A, Takahashi C, Shinose M (2001). Genome sequence of an industrial microorganism *Streptomyces avermitilis*: deducing the ability of producing secondary metabolites. Proc Natl Acad Sci USA.

[b29] Ostash B, Saghatelian A, Walker S (2007). A streamlined metabolic pathway for the biosynthesis of moenomycin A. Chem Biol.

[b30] Ostash B, Doud EH, Lin C, Ostash I, Perlstein DL, Fuse S (2009). Complete characterization of the seventeen step moenomycin biosynthetic pathway. Biochemistry.

[b31] Pethick FE, Macfadyen AC, Tang Z, Sangal V, Liu T-T, Chu J (2013). Draft genome sequence of the oxytetracycline-producing bacterium *Streptomyces rimosus* ATCC 10970. Genome Announc.

[b32] Pullan ST, Chandra G, Bibb MJ, Merrick M (2011). Genome-wide analysis of the role of GlnR in *Streptomyces venezuelae* provides new insights into global nitrogen regulation in actinomycetes. BMC Genomics.

[b33] Rückert C, Szczepanowski R, Albersmeier A, Goesmann A, Iftime D, Musiol EM (2013). Complete genome sequence of the kirromycin producer *Streptomyces collinus* Tü 365 consisting of a linear chromosome and two linear plasmids. J Biotechnol.

[b34] Rückert C, Kalinowski J, Heide L, Apel AK (2014). Draft genome sequence of *Streptomyces roseochromogenes* subsp. oscitans DS 12.976, producer of the aminocoumarin antibiotic clorobiocin. Genome Announc.

[b35] Subramaniam-Niehaus B, Schneider T, Metzger JW, Wohlleben W (1997). Isolation and analysis of moenomycin and its biosynthetic intermediates from *Streptomyces ghanaensis* (ATCC 14672) wildtype and selected mutants. Z Naturforsch [C].

[b36] Tamura K, Stecher G, Peterson D, Filipski A, Kumar S (2013). MEGA6: molecular evolutionary genetics analysis version 6.0. Mol Biol Evol.

[b37] Thomas L, Crawford DL (1998). Cloning of clustered *Streptomyces viridosporus* T7A lignocellulose catabolism genes encoding peroxidase and endoglucanase and their extracellular expression in *Pichia pastoris*. Can J Microbiol.

[b38] Toft C, Andersson SGE (2010). Evolutionary microbial genomics: insights into bacterial host adaptation. Nat Rev Genet.

[b39] Wang X-J, Yan Y-J, Zhang B, An J, Wang J-J, Tian J (2010). Genome sequence of the milbemycin-producing bacterium *Streptomyces bingchenggensis*. J Bacteriol.

[b40] Wu H, Qu S, Lu C, Zheng H, Zhou X, Bai L, Deng Z (2012). Genomic and transcriptomic insights into the thermo-regulated biosynthesis of validamycin in *Streptomyces hygroscopicus* 5008. BMC Genomics.

[b41] Xu Z, Xia J, Feng X, Li S, Xu H, Bo F, Sun Z (2014). Genome sequence of *Streptomyces albulus* PD-1, a productive strain for epsilon-poly-L-lysine and poly-L-diaminopropionic acid. Genome Announc.

[b42] Yang H, He T, Wu W, Zhu W, Lu B, Sun W (2013). Whole-genome shotgun assembly and analysis of the genome of *Streptomyces mobaraensis* DSM 40847, a strain for industrial production of microbial transglutaminase. Genome Announc.

[b43] Yang H, Zhang Z, Yan R, Wang Y, Zhu D (2014). Draft genome sequence of *Streptomyces* sp. Strain PRh5, a novel endophytic actinomycete isolated from dongxiang wild rice root. Genome Announc.

[b44] Zaburannyi N, Rabyk M, Ostash B, Fedorenko V, Luzhetskyy A (2014). Insights into naturally minimised *Streptomyces albus* J1074 genome. BMC Genomics.

[b45] Zhang H, Zhou W, Zhuang Y, Liang X, Liu T (2013). Draft genome sequence of *Streptomyces bottropensis* ATCC 25435, a bottromycin-producing actinomycete. Genome Announc.

[b46] Zhou Z, Gu J, Li Y-Q, Wang Y (2012). Genome plasticity and systems evolution in Streptomyces. BMC Bioinformatics.

